# Biological effects of naturally occurring and man-made fibres: in vitro cytotoxicity and mutagenesis in mammalian cells

**DOI:** 10.1038/sj.bjc.6690213

**Published:** 1999-03

**Authors:** R Okayasu, L Wu, T K Hei

**Affiliations:** Department of Radiation Oncology, Biology Division, Galveston, TX, 77555, USA; Center for Radiological Research, College of Physicians and Surgeons of Columbia University, New York, NY, 10032, USA

**Keywords:** chrysotile, tremolite, erionite, RCF-1, mutation, A _L_ cells

## Abstract

Cytotoxicity and mutagenicity of tremolite, erionite and the man-made ceramic (RCF-1) fibre were studied using the human– hamster hybrid A _L_ cells. Results from these fibres were compared with those of UICC Rhodesian chrysotile fibres. The A _L_ cell mutation assay, based on the *S1* gene marker located on human chromosome 11, the only human chromosome contained in the hybrid cell, has been shown to be more sensitive than conventional assays in detecting deletion mutations. Tremolite, erionite and RCF-1 fibres were significantly less cytotoxic to A _L_ cells than chrysotile. Mutagenesis studies at the *HPRT* locus revealed no significant mutant yield with any of these fibres. In contrast, both erionite and tremolite induced dose-dependent *S1*^−^ mutations in fibre-exposed cells, with the former inducing a significantly higher mutant yield than the latter fibre type. On the other hand, RCF-1 fibres were largely non-mutagenic. At equitoxic doses (cell survival at ∼ 0.7), erionite was found to be the most potent mutagen among the three fibres tested and at a level comparable to that of chrysotile fibres. These results indicate that RCF-1 fibres are non-genotoxic under the conditions used in the studies and suggest that the high mesothelioma incidence previously observed in hamster may either be a result of selective sensitivity of hamster pleura to fibre-induced chronic irritation or as a result of prolonged fibre treatment. Furthermore, the relatively high mutagenic potential for erionite is consistent with its documented carcinogenicity. © 1999 Cancer Research Campaign


*British Journal of Cancer* (1999) **79**(9/10), 1319–1324
© 1999 Cancer Research Campaign
Article no. bjoc.1998.0213

### Biological effects of naturally occurring and man-made fibres: in vitro cytotoxicity and mutagenesis in mammalian cells

**R Okayasu[1], L Wu[2] and TK Hei[2]**

[1]Department of Radiation Oncology, Biology Division, University of Texas Medical Branch, Galveston, TX 77555, USA; [2]Center for Radiological Research, College of Physicians and Surgeons of Columbia University, New York, NY 10032, USA

**Summary** Cytotoxicity and mutagenicity of tremolite, erionite and the man-made ceramic (RCF-1) fibre were studied using the human–hamster hybrid $A_L$ cells. Results from these fibres were compared with those of UICC Rhodesian chrysotile fibres. The $A_L$ cell mutation assay, based on the *S1* gene marker located on human chromosome 11, the only human chromosome contained in the hybrid cell, has been shown to be more sensitive than conventional assays in detecting deletion mutations. Tremolite, erionite and RCF-1 fibres were significantly less cytotoxic to $A_L$ cells than chrysotile. Mutagenesis studies at the *HPRT* locus revealed no significant mutant yield with any of these fibres. In contrast, both erionite and tremolite induced dose-dependent $S1^-$ mutations in fibre-exposed cells, with the former inducing a significantly higher mutant yield than the latter fibre type. On the other hand, RCF-1 fibres were largely non-mutagenic. At equitoxic doses (cell survival at ~ 0.7), erionite was found to be the most potent mutagen among the three fibres tested and at a level comparable to that of chrysotile fibres. These results indicate that RCF-1 fibres are non-genotoxic under the conditions used in the studies and suggest that the high mesothelioma incidence previously observed in hamster may either be a result of selective sensitivity of hamster pleura to fibre-induced chronic irritation or as a result of prolonged fibre treatment. Furthermore, the relatively high mutagenic potential for erionite is consistent with its documented carcinogenicity.

**Keywords:** chrysotile; tremolite; erionite; RCF-1; mutation; $A_L$ cells

The fibrogenic and carcinogenic effects of asbestos fibres are well-established. Diseases such as pulmonary asbestosis, lung cancer and malignant mesothelioma have been known to be associated with exposure to naturally occurring, as well as man-made, fibres (Merchant, 1990; Mossman et al, 1990 for review). Various in vivo and in vitro experiments have been performed in an effort to understand the pathogenic mechanisms of these fibre-induced diseases. However, no definite answer has been obtained thus far. Many in vitro studies have shown that asbestos fibres cause chromosomal aberrations (Sincock and Seabright, 1975; Jaurand, 1996 for review); however, most mutagenesis studies revealed negative results (Oshimura et al, 1984; Kelsey et al, 1986; Kenne et al, 1986). Using a unique hamster–human hybrid cell line ($A_L$), this laboratory has previously shown that both chrysotile and crocidolite fibres are highly mutagenic in mammalian cells and induce mutations in a dose-dependent manner (Hei et al, 1991, 1992, 1995). $A_L$ cells contain a whole set of hamster chromosomes as well as a single copy of human chromosome 11 that encodes a series of human cell surface antigens such as *S1*, *S2* and *S3* (Puck et al, 1971; Kao et al, 1976). Mutagenesis at the *S1* locus (M1C1 gene at 11p13) on human chromosome 11 is quantified by an antibody complement-mediated cytotoxicity assay. Since only a small portion of chromosome 11 is essential for cell viability, large-chromosomal deletions involving megabase pairs can readily be detected with the assay (Waldren et al, 1986; Hei et al, 1997).

In this report, the $A_L$ cell system was used to further investigate the mutagenic potential of tremolite, erionite and RCF-1 fibre, and the results were compared with chrysotile data previously obtained under identical experimental conditions. Tremolite has been known to be associated with a high incidence of mesothelioma cases in north-western Greece (Constantopoulos et al, 1987; Langer et al, 1987). Although tremolite was reported to be a strong inducer of micronuclei and a weak inducer of chromosomal aberrations in mammalian cells, it was non-active as a gene mutagen when tested using the bacterial *Salmonella typhimurium* assay (Athanasiou, 1992). Similarly, erionite, a fibrous zeolite with physical characteristics comparable to those of crocidolite, has been shown to be associated with pleural diseases, including mesothelioma, in Turkey (Rohl et al, 1982; Baris et al, 1987) and its high (probably the highest among various fibres) carcinogenic property has been proven in various in vivo and in vitro experiments (Maltoni et al, 1982; Johnson et al 1984; Wagner et al, 1985). A more pronounced cytogenetic effect of erionite in V79 cells as compared to either chrysotile or crocidolite fibres has been documented (Palekar et al, 1987). Furthermore, there is recent evidence to suggest that erionite induces loss of heterozygosity at the autosomal HLA-A locus in human lymphocytes (Both et al, 1994).

RCF-1 fibres have been studied less frequently than the naturally occurring fibres described above. Although the fibres are highly durable (Bellmann et al, 1994), they are less biologically

*Received 15 October 1997*
*Revised 16 September 1998*
*Accepted 22 September 1998*

*Correspondence to*: TK Hei, Center for Radiological Research, Columbia University, VC 11-218, 630 West 168th Street, New York, NY 10032, USA

reactive when compared to either crocidolite or chrysotile. RCF-1 fibres have been shown to induce modest levels of only *c-fos* and *c-jun* proto-oncogenes at high cytotoxic doses (Janssen et al, 1994) and to induce little or no anaphase/telophase abnormality in rat pleural mesothelial cells (Jaurand et al, 1994). In contrast, mesothelial cells treated with chrysotile fibres showed a much higher incidence of chromosomal abnormalities. While chronic inhalation studies showed that hamsters are highly susceptible to RCF-1, and showed an abnormally high incidence of mesothelioma (Hesterberg et al, 1991), there are examples of absence of mesothelioma yield in hamster after RCF-1 exposure. Consequently, the geometrical/dimensional factors that govern fibre ability to reach the pleura may contribute to the biological activities of fibres (Mossman and Gee, 1993 for review).

In this paper, we compare the mutagenic yield of three types of mineral fibres with that of chrysotile asbestos using the $A_L$ cell system. Two of these fibres, erionite and tremolite, are known to be carcinogenic whereas RCF-1 is currently under investigation in several laboratories. Studies performed at equitoxic doses of the fibres reveal that erionite is highly mutagenic and at a level comparable to that of chrysotiles.

### MATERIALS AND METHODS

### Cell culture

$A_L$ cells developed by Puck et al (1971) and AHI-9 cell line (a subclone of $A_L$ cells) obtained from Dr Charles Waldren were used for the studies. AHI-9 cells give identical characteristics to the parental $A_L$ cells, with a slightly lower spontaneous mutation rate. Since all the treatment for these cell lines were identical, we use $A_L$ cells for the description of cells in the rest of this paper. Normal rabbit serum was used as a source of complement, and specific monoclonal antibody against the *S1** antigen (coded by M1C1 gene on chromosome 11) was produced as described (Waldren et al, 1979). These antibodies have been shown to be highly specific for their respective human antigens and display no cross-reactivity with any hamster antigens under the conditions used in our experiments. All complement preparations were screened and those displaying high non-specific toxicity (> 30%) were rejected as described previously (Hei et al, 1988). $A_L$ cells were maintained in Ham's F-12 medium supplemented with 8% heat inactivated fetal bovine serum (Atlanta Biologicals), two times normal glycine ($2 \times 10^{-4}$ M) and 25 μm ml$^{-1}$ gentamycin. Prior to the mutagenesis assay, $A_L$ cells were cultured in HAT medium ($10^{-4}$ M hypoxanthine, aminopterin $4 \times 10^{-6}$ M and thymidine $10^{-5}$ M) for 3–4 days in order to reduce the spontaneous *HPRT*$^-$ mutants (Hei et al, 1992, 1995). Cells were used for mutation experiments within 2–3 days of treatment.

### Fibre preparation and characterization

UICC standard reference Rhodesian chrysotile was used as described (Hei et al, 1992). Tremolite from Metsovo, Greece, erionite from Rome, OR, USA and RCF-1 (TIMA standard) were obtained as gifts from Dr Robert Nolan of the Environmental Sciences Laboratory of Brooklyn College, NY, USA. Stock solutions of fibres were prepared in deionized water at concentrations ranging from 2.5 mg ml$^{-1}$ to 5.0 mg ml$^{-1}$. Briefly, samples of each fibre type were weighed out, suspended in distilled water and autoclaved to sterilize, and used at the concentrations indicated. The fibres were dispersed by sonication for 5 min before being diluted with tissue culture medium for cell treatment. For measurement of fibre size, stock solution of each fibre type was diluted tenfold with distilled water. Fifteen microlitres of the suspension were spread on a carbon disk, dried in a desiccator and then coated with 100 A of gold. The length and diameter of 80–120 fibres per sample were measured using a scanning electron microscope.

**S1* antigen is now known to be the same as the CD59 antigen.

### Determination of fibre cytotoxicity

Exponentially growing $A_L$ cells were trypsinized and plated into T-25 flasks at $1 \times 10^5$ cells per flask. Two days after plating, cells were treated with the graded doses of various fibre types (0–400 μg ml$^-$ or 0–80 μg cm$^{-2}$) in T-25 flasks with 5 ml medium for 24 h at 37°C. Following treatment, flasks were thoroughly washed with phosphate-buffered saline at least twice, trypsinized, counted and replated into 100-mm diameter petri dishes for colony formation. Cultures were incubated for 7–12 days, at which time they were fixed with formaldehyde and stained with Giemsa. The number of colonies was counted to determine the surviving fractions as described (Hei et al, 1991, 1992, 1995).

### Mutation assay

Exponential cultures exposed to various fibres for 24 h were assayed for *HPRT*$^-$, as well as *S1*$^-$, mutants for 2 consecutive weeks after the initial 1-week expression period (Hei et al, 1992). To assay for the induction of *HPRT*$^-$ mutants, $2 \times 10^5$ cells were plated into 20 100-mm dishes per dose in 10 ml complete medium containing 40 μM 6-thioguanine and incubated for 8–10 days before the mutant colonies were stained and scored. Simultaneously, at least four dishes with 200 cells per dish were plated in normal medium to determine the plating efficiency for each fibre dose. The mutant fraction at each dose ($M_F$) was determined as the number of surviving colonies divided by the total number of cells plated after correction for plating efficiency (Hei et al, 1992).

For assaying the *S1*$^-$ mutants, $5 \times 10^4$ cells were plated into each of six 60-mm dishes in 2 ml growth medium. The dishes were incubated for 2–3 h to allow cell attachment. Subsequently, 0.2% antibody and 1.5% freshly thawed complement were added to each dish. These dishes were incubated for 8–10 days before being fixed, stained and the number of *S1*$^-$ mutants scored. Controls included identical sets of dishes containing antiserum alone, complement alone, or neither agent. The mutant fraction ($M_F$) was expressed as the number of surviving colonies per $10^5$ survivors after correction for any non-specific killing due to complement alone. The mutant yield ($M_Y$) is the slope of the dose–response curve and is independent of the background mutant level.

### Statistics

Statistical analysis of data was carried out using Student's two-tailed *t*-test for unpaired data. Difference between means were regarded as significant if $P < 0.05$.

### RESULTS

The physical characterization of the various types of fibre used in the study is shown in Table 1. While the average dimensions of

 © Cancer Research Campaign 1999

**Table 1** Physical characterization of fibres used in the study

| Fibre type | Length (μm) | | Diameter (μm) | | No. of fibres per μg of sample |
|---|---|---|---|---|---|
| | a.m. ± s.d. | g.m. ± s.d. | a.m. ± s.d. | g.m. ± s.d. | |
| Chrysotile | 2.6 ± 2.4 | 1.78 ± 2.3 | 0.122 ± 0.09 | 0.12 ± 0.08 | $5.12 \times 10^6$ |
| Tremolite | 2.4 ± 3.1 | 1.41 ± 2.7 | 0.175 ± 0.13 | 0.13 ± 3.43 | $1.05 \times 10^5$ |
| Erionite | 2.2 ± 2.5 | 1.31 ± 2.9 | 0.371 ± 0.38 | 0.23 ± 2.74 | $1.27 \times 10^5$ |
| RCF-1 | 22.0 ± 25.1 | 13.5 ± 2.7 | 1.24 ± 0.78 | 0.95 ± 2.6 | $1.04 \times 10^4$ |

a.m.: arithmetic mean; g.m.: geometric mean.

chrysotile, tremolite and erionite are similar, RCF-1 fibres are substantially longer and wider than the other fibre types. Consistent with the bulkier size of the fibres, the average number of fibres per microgram of RCF-1 was also the lowest among the fibre samples examined.

The cell survival curves of exponentially growing $A_L$ cells exposed to graded doses of tremolite, erionite and RCF-1 fibres for 24 h are given in Figure 1. The curves were drawn to a linear regression model based on fitted data. All of these fibres demonstrated dose-dependent survival responses. Among the three types of fibres examined, RCF-1 was slightly more toxic than either tremolite or erionite; however, the differences were not statistically significant ($P > 0.1$). For comparison, the cell survival of $A_L$ cells exposed to graded doses of chrysotile fibres under identical experimental conditions was indicated by the dotted line in Figure 1 (Hei et al, 1992). All three fibre types were significantly less cytotoxic than chrysotiles to the $A_L$ cells. While the mean lethal dose for chrysotile was ~4 μg $cm^{-2}$ as reported previously, the corresponding values for RCF-1, tremolite and erionite were 35, 40, and 42 μg $cm^{-2}$ respectively.

Figure 2 shows mutation induction at the *HPRT* locus in $A_L$ cells treated with graded doses of the various fibres. None of these fibres induced a significant mutant yield at any of the doses examined. The spontaneous mutation yield among the $A_L$ cells used in these experiments ranged from 0.07 to 2.0 per $10^5$ survivors, and background frequency was subtracted to obtain the induced values for each dose. These data were similar to those of chrysotile, which showed no statistically significant mutation yield over a range of fibre doses examined (Hei et al, 1992). The results were consistent with other published reports on mutation induction by mineral fibres at this gene locus (Oshimura et al, 1984; Kelsey et al, 1986; Kenne et al, 1986).

Since previous studies from this laboratory demonstrated a significant *S1*⁻ mutant induction in $A_L$ cells exposed to both chrysotile and crocidolite fibres (Hei at al, 1991, 1992), similar experiments were performed with RCF-1, erionite and tremolite as

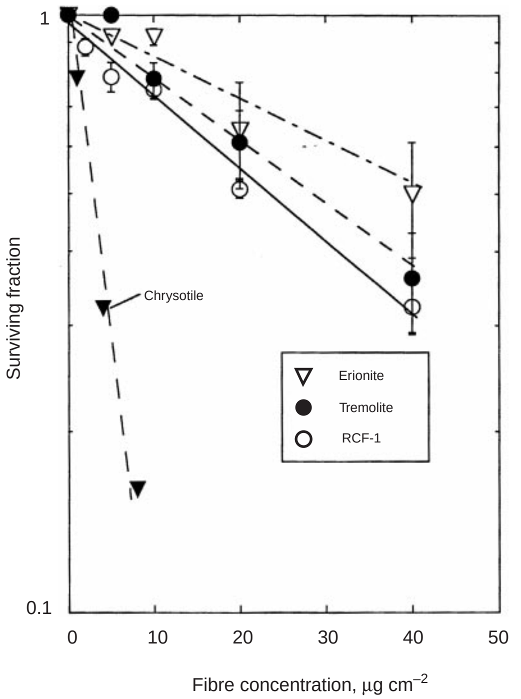

**Figure 1** Dose–response survival of $A_L$ cells treated with graded doses of erionite, tremolite and RCF-1 fibres for 24 h. Cell survival with chrysotile fibres obtained under identical treatment conditions is given for comparison. The curves represent best fit of the data from 3–6 independent experiments. Bars represent ± S.E.M.

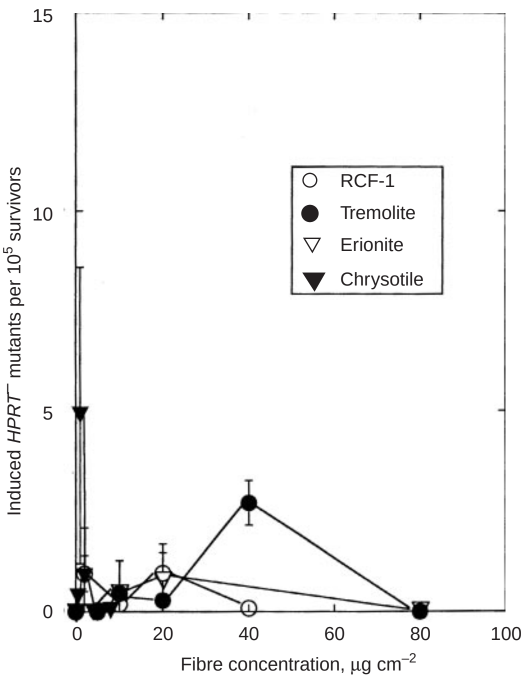

**Figure 2** Induced mutant fraction at the *HPRT* locus in $A_L$ cells exposed to RCF-1, tremolite, erionite and chrysotile fibres. Cells were exposed to graded doses of the fibres for 24 h and assayed for mutation for 2 consecutive weeks after the initial 7-day expression period. Induced mutant fraction = total mutant yield minus pre-existing background mutants. Data are pooled from 2–4 independent experiments. Bars represent ± S.E.M.

© Cancer Research Campaign 1999

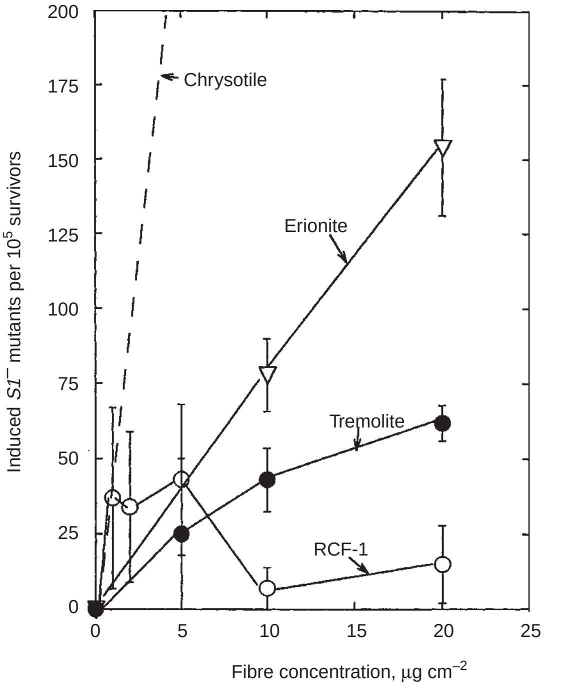

**Figure 3** Induced mutant fractions at the *S1* locus by graded doses of the various fibres. Data are pooled from 2–4 independent experiments. Bars represent ± S.E.M.

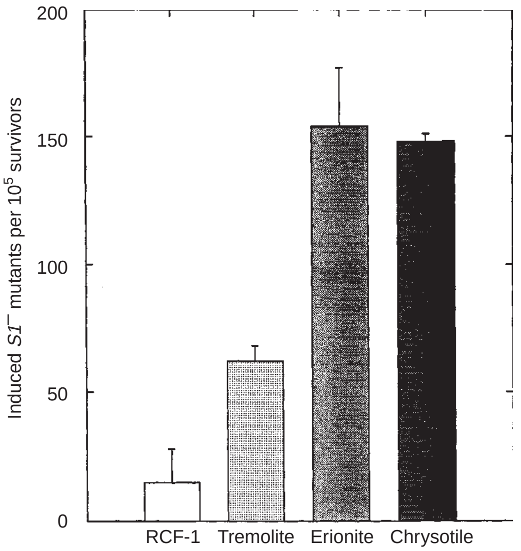

**Figure 4** Induced mutant fractions at the *S1* locus by equitoxic doses of the various fibres (survival fraction ~0.7). The fibre doses used for each fibre are 10 µg cm$^{-2}$ for RCF-1, 20 µg cm$^{-2}$ for both tremolite and erionite, and 2 µg cm$^{-2}$ for chrysotile fibres shown in Figure 3. Mutation data were pooled from three to four independent experiments and the fraction of pre-existing $S1^-$ mutants in the $A_L$ cells population used in these experiments ranged from 65 to 94 per $10^5$ survivors. Both erionite and tremolite fibres revealed clear dose–response mutant induction for concentrations up to 20 µg cm$^{-2}$. In contrast, RCF-1 fibres were much less effective in mutant induction throughout the range of doses examined. Although the number of induced $S1^-$ mutants in some dose groups were greater than zero, the difference in the mutant fraction between the highest and lowest doses was not statistically significant ($P > 0.5$) so that pooled data from four experiments produced no consistent dose–response for the yield of mutants at that locus. As a function of fibre concentration, chrysotile was the most mutagenic of all the fibres examined. Since a mutation only manifests in living cells, it is more relevant to compare the mutagenic potential of the different fibres at doses that result in equivalent survival level. Figure 4 compares the induced mutation frequencies of the various fibres with that of chrysotiles at doses that resulted in a survival fraction of ~0.7. Here the mutagenic potential of erionites was comparable to that of chrysotiles, and the mutant fraction was two- and tenfold higher than that of tremolite and RCF-1 fibres respectively.

### DISCUSSION

Several conclusions can be drawn from the present studies. First of all, erionite, tremolite and RCF-1 fibres are less cytotoxic to mammalian cells than chrysotile fibres on a per unit weight basis under the conditions used in the present studies. Secondly, no significant induction of $HPRT^-$ mutants is detected in $A_L$ cells exposed to any of these fibres. On the other hand, a dose-dependent mutation yield is observed at the *S1* locus in cells exposed to either erionite or tremolite fibres, as in the case with both chrysotile and crocidolite fibres. Although the exact mechanism responsible for this discrepancy is not known, the results are consistent with our previous reports that mutants induced by asbestos fibres are poorly recovered at the *HPRT* locus because they are lethal (Hei et al, 1991, 1992, 1995). In contrast, since only a small part of region 11p15.5 is required for the viability of $A_L$ cells, mutations in the human chromosome 11 ranging in size up to 140 million bases of DNA can be detected. These results reaffirm the sensitivity of the $A_L$ cell system for identifying mutagens that induce predominately multilocus deletions, including radon $\alpha$ particles (Evans, 1993; Zhu et al, 1996; Hei et al, 1997) and arsenic (Hei et al, 1998).

The strong mutagenic potential of erionite, as demonstrated in this report, is consistent with its carcinogenic effects demonstrated in both epidemiological and animal studies (Maltoni et al, 1982; Rohl et al, 1982; Johnson et al, 1984; Wagner et al, 1985). Although the carcinogenic mechanism of erionite is not clear, there is evidence to suggest that its ability to induce hydroxyl radicals may be critical to its genotoxic effects in vitro (Maples and Johnson, 1992). The origin of reactive oxygen species (ROS) in fibre-treated cells is not clear. Several mechanisms have been proposed for the generation of ROS by asbestos. First, fibres per se trigger ROS production by iron-catalysed Fenton reactions (Aust, 1994), and second, fibres may activate phagocytosis to enhance the production of ROS (Mossman and Gee, 1993). It has been postulated that 'frustrated phagocytosis' of long fibres is a potent stimulus for ROS production in macrophages and leucocytes. However, the observations that non-iron-containing fibres, such as erionites, are carcinogenic (Baris et al, 1987) suggest that the mobilization of iron from fibres may not be the only pathway for ROS induction.

The large difference in cell survival after chrysotile treatment versus exposure to the other fibres examined may be attributed either to differences in total fibre number per unit weight or in the

 *© Cancer Research Campaign 1999*

way cells handle these fibres. All of the fibres examined, including RCF-1 fibres, were found to be internalized by $A_L$ cells to various extents. On a per unit weight basis, there are significantly more chrysotile than RCF-1 fibres. However, it should be noted that in vitro cytotoxicity, while being a useful end point for comparison, may have little or no relevance to in vivo biological responses to mineral fibres (Yeagles et al, 1995). In vitro cytotoxicity is clearly dependent on fibre length. As mentioned by Jaurand et al (1994), rat mesothelial cells treated with RCF-1 fibres phagocytized a much lower number of Stanton’s fibres than cells treated with chrysotile. Differences in surface morphology and fibre dimension are important modulating factors in fibre toxicology. In general, long and thin fibres are known to be more biologically active (e.g. Stanton et al, 1977). Although RCF-1 fibres are significantly longer than chrysotile fibres as shown in Table 1, they are much larger in diameter and, as a result, both the surface area and the number of fibres per unit weight of sample are smaller than either for chrysotile or erionite preparations. Furthermore, based on dimensional measurements, the amount of amorphous materials in our RCF-1 samples is ~18%. The large internal surface area of erionite, for example, is essential for mesothelioma induction by binding to free iron and promoting oxyradical formation (Coffin and Ghio, 1991). While the size distribution of RCF-1 is significantly larger than erionite or tremolite, the survival levels induced in $A_L$ cells by graded doses of these fibres are similar. Previous studies have shown that the number of fibres required to achieve a mean lethal dose in V79 cells for various fibre types differ by an order of magnitude (Palekar et al, 1987). The observation that asbestos fibres are commonly found internalized in $A_L$ cells that undergo mutational changes (Hei et al, 1991) indicates the importance of fibre–cell interaction in asbestos-mediated genotoxicity.

How could the negative mutagenesis data with RCF-1 fibres explain the high incidence of mesothelioma in hamsters as previously reported in some studies? First of all, there is the species factor since Syrian golden hamsters in the Hesterberg study may be more sensitive to ceramic fibres than other species, such as rats (Hesterberg et al, 1991). There is recent evidence to suggest that hamster pleura is a more sensitive target organ for fibre-induced disease than is the pleura of the rat, and that hamsters develop more severe fibrotic changes and mesothelial cell proliferation in the pleura than do rats (Everitt et al, 1997). Secondly, there is the chronic versus acute exposure factor. In the present studies, a treatment period of 24 h was chosen so that the results would be comparable to our previous data collected under identical experimental conditions using the same assay system. Obviously, prolonged exposure to RCF-1 would be necessary for mesothelioma induction to occur in animals. Furthermore, there may be a threshold RCF-1 dose necessary to initiate the transformation process, as previously suggested using anaphase/telophase abnormality as an end point (Yegles et al, 1995). Since a long latency period is often associated with fibre-induced cancer in general, the importance of fibre durability in cells should be further emphasized.

### ACKNOWLEDGEMENTS

The authors thank Dr Ann-Judith Silverman of the Department of Anatomy and Cell Biology at Columbia University for her invaluable help in electron microscopy. Thanks are also due to Ms Xu An for her help in the fibre dimensional analysis. This work was supported in part by the National Institute of Environmental Health Sciences grants ES 05876 and ES06831 (TKH), and the National Cancer Institute grant CA 71438 (RO).

### REFERENCES

Athanasiou K, Constantopoulos SH, Rivedal E, Fitzgerald DJ and Yamasaki H (1992) Mestovo-tremolite asbestos fibers: in vitro effects on mutation, chromosome aberration, cell transformation and intercellular communication. *Mutagenesis* **7**: 343–347

Aust A (1994) The role of iron in asbestos induced cancer. In *Cellular and Molecular Effects of Mineral and Synthetic Dusts and Fibers*, NATO ASI Series, Vol. H 85, Davis JMG and Jaurand M-C (eds) pp. 53–61. Springer-Verlag: Berlin

Baris YI, Simonato L, Artvinli M, Pooley F, Saracci R, Skidmore J and Wagner C (1987) Epidemiological and environmental evidence of the health effects of exposure to erionite fibers: a four-year study in the Cappadocian region of Turkey. *Int J Cancer* **39**: 10–17

Bellman B, Muhle H and Pott F (1994) Investigation of the bio-durability and carcinogenicity of different man-made mineral fibers. In *Cellular and Molecular Effects of Mineral and Synthetic Dusts and Fibers*, NATO ASI Series, Vol. H 85, Davis JMG and Jaurand M-C (eds), pp. 299–304. Springer-Verlag: Berlin

Both K, Henderson DW and Turner DR (1994) Asbestos and erionite fibers can induce mutations in human lymphocytes that result in loss of heterozygosity. *Int J Cancer* **59**: 538–542

Coffin DL and Ghio AJ (1991) Relative intrinsic potency of asbestos and erionite fibers: proposed mechanism of action. In *Mechanisms of Fibre Carcinogenesis*, NATO ASI series, Vol. A223, Brown RC, Hoskins JA, and Johnson NF (eds), pp. 71–80. Plenum Press: New York

Constantopoulos SH, Malamou-Mitsi VD, Gaudevenos JA, Papathanasiou MP, Pavlidis NA and Papadimitriou CS (1987) High incidence of malignant pleural mesothelioma in neighboring villages of northwestern Greece. *Respiration* **51**: 266–271

Evans HH (1993) Cellular and molecular effects of radon and other alpha particle emitters. *Adv Mutat Res* **3**: 28–52

Everitt JI, Gelzleichter TR, Bermudez E, Mangum JB, Wong BA, Janszen DB and Moss OR (1997) Comparison of pleural responses of rats and hamsters to subchronic inhalation of refractory ceramic fibers. *Environ Health Perspect* **105**: 1209–1214

Hei TK, Hall EJ and Waldren CA (1988) Mutation induction by neutrons as determined by an antibody complement mediated cell lysate system. I. Experimental observation. *Radiat Res* **115**: 281–291

Hei TK, He ZY, Piao CQ and Waldren C (1991) The mutagenicity of mineral fibers. In *Mechanisms in Fiber Carcinogenesis*, Brown RC et al. (eds), pp. 319–325. Plenum Press: New York

Hei TK, Piao CQ, He ZY, Vannais D and Waldren CA (1992) Chrysotile fiber is a strong mutagen in mammalian cells. *Cancer Res* **52**: 6305–6309

Hei TK, He ZY and Suzuki K (1995) Effects of antioxidants on fiber mutagenesis. *Carcinogenesis* **16**: 1573–1578

Hei TK, Wu LJ, Liu SX, Vannais D, Waldren CA and Randers-Pehrson G (1997) Mutagenic effects of a single and an exact number of alpha particles in mammalian cells. *Proc Natl Acad Sci USA* **94**: 3765–3770

Hei TK, Liu SX and Waldren CA (1998) Mutagenicity of arsenic in mammalian cells: role of reactive oxygen species. *Proc Natl Acad Sci USA* **95**: 8103–8107

Hesterberg TW, Mast R, McConnel EE, Chevalier J, Bernstein WBB and Anderson R (1991) Chronic inhalation toxicity of refractory ceramic fibers in Syrian hamsters. In *Mechanisms in Fiber Carcinogenesis*, Brown RC et al (eds), pp. 531–538. Plenum Press: New York

Janssen YMW, Heintz NH, Marsh JP, Borm PJA and Mossman BT (1994) Induction of *c-fos* and *c-jun* proto-oncogenes in target cells of the lung and pleura by carcinogenic fibers. *Am J Respir Cell Mol Biol* **11**: 522–530

Jaurand MC (1996) Use of in vitro genotoxicity and cell transformation assays to evaluate the potential carcinogenicity of fibres. In *Mechanisms of Fibre Carcinogenesis*, Kane AB, Boffetta P, Saracci R and Wilbourn JD (eds), pp. 55–72. IARC 140: Lyon

Jaurand MC, Yeagles M, Dong HY, Renier A, Saint Etienne L, Kheuang L, Jason X and Bignon J (1994) In vitro DNA and chromosome damage produced by some minerals and man made particles on rat pleural mesothelial cells (RPMC): mechanisms and relationship with in vivo experimental findings. In *Cellular*

*© Cancer Research Campaign 1999* 

*and Molecular Effects of Mineral and Synthetic Dusts and Fibers*, NATO ASI Series, vol. H85, Davis JMG and Jaurand M-C (eds), pp. 183–192. Spring-Verlag: Berlin.

Johnson NF, Edwards RE, Munday DE, Rowe N and Wagner JC (1984) Pluripotential nature of mesothelioma induced by inhalation of erionite in rats. *Br J Exp Pathol* **65**: 377–388

Kao FT, Jones CC and Puck T (1976) Genetics of somatic mammalian cells: genetic, immunologic and biochemical analysis with CHO cells hybrids containing selected human chromosomes. *Proc Natl Acad Sci USA* **68**: 3102–3106

Kelsey KT, Yano E, Lieber HL and Little JB (1986) The in vitro effects of fibrous erionite and crocidolite asbestos. *Br J Cancer* **54**: 107–114

Kenne K, Lingquist S and Ringertz NR (1986) Effects of asbestos fibers on cell division, survivals, and formation of thioguanine resistant mutants in CHO cells. *Environ Res* **39**: 448–464

Langer AM, Nolan RP, Constantopoulos SH and Moutsopoulos HM (1987) Association of Metsovo lung and pleural mesothelioma with exposure to tremolite containing whitewash. *Lancet* **i**: 965–967

Maltoni C, Minardi F and Morisi L (1982) Pleural mesothelioma in Sprague-Dawley rats by erionite: first experimental evidence. *Environ Res* **28**: 238–244

Maples KR and Johnson NF (1992) Fiber-induced hydroxyl radical formation: correlation with mesothelioma induction in rats and humans. *Carcinogenesis* **13**: 2035–2039

Merchant JA (1990) Human epidemiology: a review of fiber type and characteristics in the development of malignant and nonmalignant disease. *Environ Health Perspect* **88**: 287–293

Mossman BT and Gee BL (1993) Pulmonary reactions and mechanisms of toxicity of inhaled fibers. In *Toxicology of the Lung*, 2nd Edn, Gardner et al (eds), pp. 371–387. Raven Press: New York

Mossman BT, Bignon J, Corn M, Seaton A and Gee BL (1990) Asbestos: scientific development and implications for public policy. *Science* **249**: 249–300

Oshimura M, Hesterberg T, Tsutsui T and Barrett JC (1984) Correlation of asbestos induced cytogenetic effects with cell transformation of Syrian hamster embryo cells in culture. *Cancer Res* **44**: 5017–5022

Palekar LD, Eyre JF, Most BM and Coffin DL (1987) Metaphase and anaphase analysis of V79 cells exposed to erionite, UICC chrysotile and UICC crocidolite. *Carcinogenesis* **8**: 553–560

Puck TT, Wuchier P, Jones C and Kao FT (1971) Genetics of somatic mammalian cells: lethal antigens and genetic markers for study of human linkage groups. *Proc Natl Acad Sci USA* **68**: 3102–3106

Rohl AN, Langer AM, Moncure G, Selikoff IJ and Fischbein A (1982) Endemic pleural disease associated with exposure to mixed fibrous dust in Turkey. *Science* **216**: 518–520

Sincock A and Seabright M (1975) Induction of chromosome changes in Chinese hamster cells by exposure to asbestos fibers. *Nature* **257**: 56–58

Stanton MF, Layard M, Tegeris A, Miller E, May M and Kent E (1977) Carcinogenicity of fibrous glass: pleural response in the rat in relation to fiber dimension. *J Natl Cancer Inst* **58**: 587–597

Wagner JC, Skidmore JW, Hill RJ and Griffiths DM (1985) Erionite exposure and mesothelioma in rats. *Br J Cancer* **51**: 727–730

Waldren C, Jones CC and Puck TT (1979) Measurement of mutagenesis in mammalian cells. *Proc Natl Acad Sci USA* **76**: 1358–1362

Waldren C, Correll L, Sognier MA and Puck TT (1986) Measurement of low levels of X-ray mutagenesis in relation to human disease. *Proc Natl Acad Sci USA* **83**: 4839–4843

Yegles M, Janson X, Dong HY, Renier A and Jaurand MC (1995) Role of fibre characteristics on cytotoxicity and induction of anaphase/telophase aberrations in rat pleural mesothelial cells in vitro: correlation with in vivo animal findings. *Carcinogenesis* **16**: 2751–2758

Zhu LX, Waldren CA, Vannais D and Hei TK (1996) Cellular and molecular analysis of mutagenesis induced by charged particles of defined LET. *Radiat Res* **145**: 251–259

 *© Cancer Research Campaign 1999*
